# Associations between postprandial symptoms, hydrogen and methane production, and transit time in irritable bowel syndrome

**DOI:** 10.1111/nmo.14482

**Published:** 2022-10-11

**Authors:** Joost P. Algera, Esther Colomier, Chloé Melchior, Jóhann P. Hreinsson, Irina Midenfjord, Egbert Clevers, Magnus Simrén, Hans Törnblom

**Affiliations:** ^1^ Department of Molecular and Clinical Medicine, Institute of Medicine, Sahlgrenska Academy University of Gothenburg Gothenburg Sweden; ^2^ Department of Chronic Diseases, Metabolism and Aging (CHROMETA), Translational Research Center for Gastrointestinal Disorders (TARGID) KU Leuven Leuven Belgium; ^3^ Gastroenterology Department and INSERM CIC‐CRB 1404 Rouen University Hospital Rouen France; ^4^ INSERM U1073 Rouen Normandy University Rouen France; ^5^ Centre for Functional GI & Motility Disorders University of North Carolina at Chapel Hill Chapel Hill North Carolina USA

**Keywords:** colonic fermentation, gastrointestinal transit, IBS, symptom assessment, visceral hypersensitivity

## Abstract

**Background:**

Abnormal oroanal transit time (OATT) and visceral hypersensitivity are key pathophysiological factors in irritable bowel syndrome (IBS). The lactulose nutrient challenge test (LNCT) has been developed to assess the postprandial symptoms and gut microbial fermentation. We aimed to investigate associations between OATT, rectal sensitivity, and LNCT in IBS patients.

**Methods:**

We included 263 IBS patients from two study cohorts, where the link between pathophysiology and symptoms was investigated. During the LNCT, severity of postprandial symptoms was graded, and breath hydrogen/methane concentrations were measured after ingestion of a combined lactulose nutrient drink every 15 min for 4 h. The patients underwent rectal sensitivity (rectal barostat) and OATT (radiopaque markers) investigations. Comorbid conditions (functional dyspepsia, anxiety, depression, and somatization) were assessed with questionnaires.

**Key Results:**

After controlling for comorbid conditions, rectal sensitivity was associated with abdominal pain (*p* < 0.05), and more rapid OATT was associated with higher severity of abdominal discomfort, rumbling, nausea, and urgency (*p* < 0.05 for all) both pre‐ and post‐prandially. Postprandial nausea, urgency, and abdominal pain changed differently over time depending on OATT (*p* < 0.05 for all). OATT, but not rectal sensitivity, was associated with hydrogen and methane concentrations (*p* = 0.002 for both). Trajectories over time of postprandial symptoms and exhaled hydrogen/methane concentrations were correlated with different correlations depending on OATT.

**Conclusion and Inferences:**

This study highlights the importance of oroanal transit and hydrogen and methane production in the pathophysiology of IBS and increases our understanding of pathophysiological factors involved in postprandial symptom generation. Treatments targeting oroanal transit and hydrogen and methane production may improve specific postprandial symptoms.

## Graphical abstract

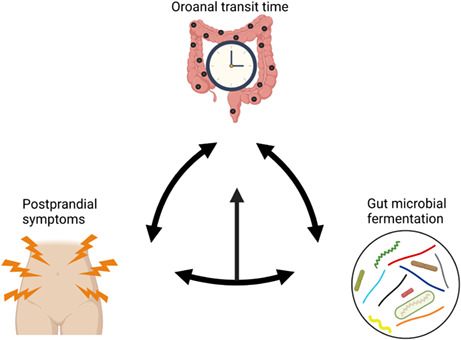

**Key Points**

- Postprandial symptoms, hydrogen and methane production, and oroanal transit time are reciprocally associated in irritable bowel syndrome, with different associations depending on transit time.
- Oroanal transit time and hydrogen and methane production may be relevant treatment targets for reduction of postprandial symptoms in irritable bowel syndrome.

## 1 INTRODUCTION

Irritable bowel syndrome (IBS) is characterized by recurrent abdominal pain that is associated with altered bowel habits.^1^ Abnormal gut‐brain interactions, psychological distress, visceral hypersensitivity, altered gastrointestinal (GI) motility, and gut microenvironment alterations are all considered as pathophysiological factors in IBS,^2^, ^3^, ^4^, ^5^, ^6^, ^7^ but detailed understanding of this multifactorial pathophysiology is lacking.

Many IBS patients report that food items trigger their GI symptoms, indicating that postprandial mechanisms are of key importance in GI symptom generation.^4^ Despite this, few studies have investigated postprandial symptoms in IBS. Our group developed a measure to study the symptomatic effect of nutrient intake, which reflects the overall IBS symptom severity and visceral sensitivity, as well as gut microbial fermentation, including measures of hydrogen and methane production: the lactulose nutrient challenge test (LNCT).^8^ Previous studies, assessing the LNCT and a meal challenge test,^9^, ^10^ found that depression, anxiety, somatization, and comorbid functional dyspepsia (FD) influence postprandial symptom patterns in IBS.

Other pathophysiological abnormalities may also be associated with the generation of postprandial symptoms, including visceral hypersensitivity and oroanal transit time (OATT). Visceral hypersensitivity, that is, increased intensity of visceral sensations or a decreased pain threshold for visceral stimuli, is known to be associated with overall IBS symptom severity,^5^, ^11^ but a clear link with postprandial symptoms is lacking. Abnormal OATT is reported to be associated with bowel habit, IBS subtype, and to some extent with IBS symptoms in general. However, the link between OATT and overall IBS symptoms remains debated.^6^, ^12^, ^13^ Intestinal gases, produced by the gut microbiota through fermentation of carbohydrates, can affect gut physiology and are proposed to be involved in the generation of GI symptoms, but there is lack of evidence regarding this.^14^, ^15^, ^16^ Currently, there are no data available, based on a large number of patients, on the link between postprandial symptoms, hydrogen and methane production, and other pathophysiological factors in IBS.

In this study, we hypothesized that OATT, rectal sensitivity, and hydrogen and methane production are associated with the severity of postprandial symptoms in IBS. Therefore, we aimed to investigate associations between postprandial symptoms and (1) OATT as measured by radiopaque markers, (2) rectal sensitivity as measured by rectal barostat, and (3) hydrogen and methane production as measured by breath test in IBS, while controlling for anxiety, depression, somatization, and FD. Additionally, we aimed to investigate if hydrogen and methane production is linked with OATT and rectal sensitivity.

## 2 MATERIALS AND METHODS

### 2.1 Participants

Adult IBS patients from two prospective cohort studies, assessing the relevance of different pathophysiological factors for symptom generation in IBS, were included between 2010 and 2015 (cohort 1), and between 2016 and 2019 (cohort 2) at our specialized unit for neurogastroenterology at the Sahlgrenska University Hospital, Gothenburg, Sweden. IBS and comorbid FD were defined by the Rome III^17^ or Rome IV^1^ criteria, in accordance with diagnostic standards at time of inclusion. IBS patients that completed the LNCT were included. Exclusion criteria were severe cardiovascular, hepatic or neurological diseases, psychiatric disease being the dominant clinical problem, other gastrointestinal (GI) disease explaining the GI symptoms, diabetes, GI surgery (except for appendectomy or cholecystectomy), use of antibiotics within 1 month before initiation of the study, and pregnant or lactating females. Celiac disease and inflammatory bowel disease were excluded by a thorough clinical history and by routine laboratory tests, including tissue transglutaminase antibodies, C‐reactive protein (CRP) and fecal calprotectin, respectively. Further examinations, for example, endoscopies, were initiated as part of the clinical routine if judged to be indicated by the gastroenterologist (MS and HT). Medications affecting GI function were not allowed for 2–7 days (depending on the medication), before and during the investigations. The patients gave written and verbal informed consent before any procedures were performed, and the Regional Ethical Review Board in Gothenburg approved the studies (731/09, 988/14) according to the Declaration of Helsinki. The planning of the study‐related procedures for both cohorts is presented in Figure S1. All authors had access to the study data and reviewed and approved the final manuscript.

### 2.2 Lactulose nutrient challenge test

After an overnight fast (≥8 h), each patient arrived at our laboratory and underwent the combined lactulose (25 g) and nutrient (400 ml Fortimel Energy, 600 kcal, 49% carbohydrates, 35% fat, 16% protein; gluten‐free and lactose‐free) challenge test.^8^ The severity of eight GI symptoms, that is, gas, nausea, urgency, abdominal bloating, discomfort, distension, rumbling, and pain, were graded on Likert scales (0–20). Pre‐prandial symptoms were assessed at baseline and postprandial symptoms were assessed every 15 min at 16 time points (15–240 min). Higher scores indicated more severe symptoms. The severity of the assessed symptoms reflects the severity of daily symptoms in IBS patients.^8^ Exhaled hydrogen and methane concentrations were also measured at 15‐min intervals as an indirect biomarker of gut microbial fermentation (QuinTron Breath Tracker; QuinTron Instrument Company).

### 2.3 Oroanal transit time

The OATT was assessed in both cohorts using our extensively validated method.^6^, ^18^, ^19^, ^20^ The patients were instructed to ingest 10 radiopaque markers in the morning for five consecutive days, and five markers ingested in the morning, and five markers in the evening on the sixth day. On the morning on the seventh day, the patients underwent fluoroscopy where the radiopaque markers remaining in the gut were counted. OATT expressed in days are given by dividing number of remaining markers by 10. Reference values (5th and 95th percentile) from healthy controls in previous studies at our laboratory were used to identify abnormal values, that is, delayed and accelerated OATT (normal OATT: females 0.9–3.9 days; males 0.7–2.1 days).^6^, ^18^, ^19^, ^20^

### 2.4 Rectal sensitivity

IBS patients in cohort 1 underwent a rectal barostat procedure, investigated with a polyethylene balloon attached to a double lumen polyvinyl tube (Salem Sump Tube, 18F; Sherwood Medical) with 8 cm distance between the balloon attachment sites allowing inflation to a maximal volume of 650 ml, spherical balloon shape. The catheter was connected to a computer driven electronic barostat (Dual Drive Barostat, Distender Series II; G&J Electronics Inc.) that controlled the inflation. After a habituation sequence with 4 mmHg increments every 15 s until 20 mmHg or the discomfort threshold, the ramp inflation started from 0 mmHg and with 4 mmHg increments every minute until the subjects reported pain or a maximum pressure of 60 mmHg was reached. The pain threshold (mmHg) represented maximal tolerable inflation and was used as our measure of rectal sensitivity in cohort 1. To identify patients with rectal hypersensitivity, we used normal values (5th and 95th percentile) from healthy volunteers (pain threshold <20 mmHg).^5^

### 2.5 Questionnaires

The severity of GI symptoms during the past week was assessed by use of the Gastrointestinal Symptom Rating Scale (GSRS)‐IBS,^21^ which includes 13 items rated from 1 (no discomfort at all) to 7 (very severe discomfort) on Likert scales total score range (13–91). Five subscales can be evaluated by combination of specific responses; diarrhea syndrome, constipation syndrome, satiety, bloating syndrome, and pain syndrome.^21^

The Bristol Stool Form Scale (BSFS),^22^ which characterizes seven different stool form types (range 1–7, hard‐loose), was registered in a diary for 14 days to categorize subjects into three IBS subtypes according to the Rome IV definition of subtypes: IBS with constipation (IBS‐C), IBS with diarrhea (IBS‐D), and IBS without predominant diarrhea or constipation (IBS‐nonCnonD) that include IBS with mixed bowel habits and unclassified IBS.^22^ The diary data were also used to define average stool frequency, and average stool consistency.

Anxiety and depression were screened using the Hospital Anxiety and Depression scale (HADS),^23^ which measures reported symptoms of anxiety and depression (both seven items) during the last week on two subscales. The items are scored from 0 to 3, higher scores indicate more severe psychological distress in the respective subscale (score range: 0–21 per subscale).^23^

A modified version of the Patient Health Questionnaire (PHQ)‐15, where the three questions assessing GI symptoms are excluded to solely assess non‐GI somatic symptoms, the PHQ‐12^24^ was used as a measure of somatization. It contains 12 items scored from 0 to 2, where a higher score indicated higher symptom severity (score range: 0–24 for women; 0–22 for men where the menstrual problems question was excluded).^24^

### 2.6 Data analysis

To confirm the associations previously found between postprandial symptoms and anxiety, depression, somatization, and FD after a meal challenge test and LNCT,^9^, ^10^ we used linear mixed models with heterogeneous autoregressive covariance pattern to assess the data. The models included main effects of time (categorical variable, 17 time points), the variable of interest (anxiety, depression, somatization [continuous variables], and FD [dichotomous variable]), and variable of interest‐by‐time interaction effect. The variables of interest were assessed in separate models and outcome variables were the postprandial symptoms (log‐transformed). A significant main effect of a variable of interest indicated an association between the variable of interest and the dependent variable, which is independent of time. A significant variable of interest‐by‐time interaction effect indicated that the dependent variable changed differently over time depending on the variable of interest. The next step was to test the hypothesis that OATT and rectal sensitivity were associated with postprandial symptoms and hydrogen and methane production. Here, OATT and rectal sensitivity (continuous variables) were modeled as described above (unadjusted and adjusted for comorbid conditions). Outcome variables were postprandial symptoms and exhaled hydrogen/methane concentrations. For visualization, the associations were plotted over time (estimated marginal means) at different levels of the continuous variables of interest (<25th; 25th–75th; >75th percentile), with anxiety, depression, and somatization held constant at the mean of the total population. Finally, we assessed the role of hydrogen and methane production in postprandial symptom generation using dynamical correlations (unadjusted for comorbid conditions). Dynamical correlations assessed if exhaled hydrogen/methane and postprandial symptoms followed a common trajectory over time within a patient, which may indicate a relationship. The dynamical correlations were expressed as within‐person correlations (WPC) with T and p‐values, both controlled for regular between‐person (Pearson) correlations. Interpretation of the dynamical correlations was done by the combination of WPC (0–1.0) and *p*‐values. Strong WPC in combination with *p* > 0.05 are irrelevant, as well as low *p*‐values in combination with weak WPC (<0.2). See Appendix S1 for more details. SPSS statistics version 27.0 (SPSS) and RStudio (R version 4.0.3) were used to analyze the data, and *p*‐values <0.05 were considered as statistically significant.

## 3 RESULTS

### 3.1 Participants

A total of 263 IBS patients were included: 143 from cohort 1 (Rome III) and 120 from cohort 2 (Rome III, *n* = 84; Rome IV, *n* = 36). Age, sex, and IBS subtype distribution, comorbid FD, and other relevant clinical information did not differ substantially between the cohorts (Table 1). No multicollinearity (*r* > 0.7) was found among the independent variables (Table S1).

**TABLE 1:** Demographics and clinical characteristics

	Cohort 1 (*n* = 143)	Cohort 2 (*n* = 120)	*p*‐Value^a^	Study population (*n* = 263)
Age, years	34.9 ± 11.2	36.7 ± 12.5	0.21	35.7 ± 11.8
Female sex, %	65	68	0.57	67
BMI, kg/m^2^	23.4 ± 3.6	23.8 ± 4.5	0.44	23.6 ± 4.0
Subtype, %				
IBS‐C	28	35	0.26	31
IBS‐D	37	40		38
IBS‐nonCnonD	35	25		31
Stool frequency, *n*/day	1.9 ± 1.3	1.9 ± 1.0	0.98	1.9 ± 1.1
Stool consistency, BSFS	4.2 ± 1.2	4.0 ± 1.1	0.31	4.1 ± 1.2
Comorbid FD, %	44	46	0.72	45
GSRS‐IBS total	3.7 ± 0.9	3.9 ± 0.6	0.05	3.8 ± 0.9
HADS anxiety	8.5 ± 4.5	7.6 ± 4.6	0.11	8.1 ± 4.6
HADS depression	5.3 ± 3.6	5.7 ± 3.6	0.40	5.5 ± 3.6
PHQ‐12 somatization	8.2 ± 4.2	7.6 ± 3.7	0.24	7.9 ± 4.0
Rectal sensitivity, mmHg	27.7 ± 8.8	NA		
Rectal hypersensitivity, %	27	NA		
OATT, days	1.4 ± 1.1	1.6 ± 1.1	0.21	1.5 ± 1.1
Delayed transit, %	6	10	0.08	9
Normal transit, %	66	73		68
Accelerated transit, %	28	17		23

Abbreviations: BMI, body mass index; BSFS, Bristol Stool Form Scale; FD, functional dyspepsia; GSRS, gastrointestinal symptom rating scale, HADS, hospital anxiety and depression scale; IBS, irritable bowel syndrome; IBS‐C, IBS with constipation; IBS‐D, IBS with diarrhea; IBSnonCnonD, IBS with mixed bowel habits or unclassified IBS; NA, not applicable; OATT, oroanal transit time; PHQ, patient health questionnaire.

^a^ Student *T*‐test or *χ* ^2^ test (cohort 1 vs. cohort 2).

### 3.2 Comorbid conditions are associated with pre‐ and postprandial GI symptoms in IBS

As previously reported,^9^, ^10^ higher levels of anxiety, depression, and somatization, and presence of FD were found to be associated with several pre‐ and post‐prandial GI symptoms (Table S2). Anxiety was positively associated with severity of GI symptoms, (except for urgency). This was similar for higher levels of depression (except for gas and abdominal rumbling) and somatization (except for gas, abdominal rumbling, and urgency). The presence of FD was also associated with the severity of postprandial symptoms (except for abdominal rumbling and urgency), with higher postprandial symptom ratings for IBS patients with comorbid FD (Table S2).

### 3.3 OATT is associated with intensity of GI symptoms and specific postprandial symptom responses

**FIGURE 1:**
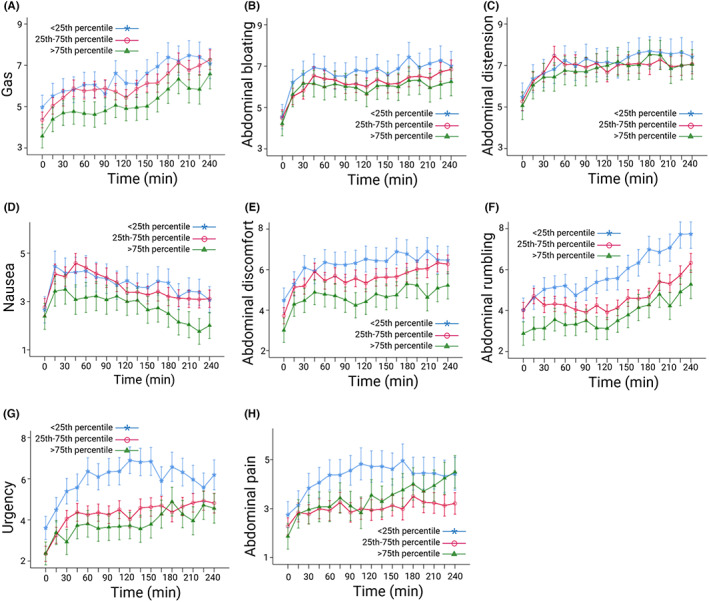
Associations between OATT and postprandial symptom ratings in IBS patients (*n* = 254). The curves are estimated marginal means with standard error of the mean. (A) Gas, (B) abdominal bloating, (C) abdominal distension, (D) nausea, (E) abdominal discomfort, (F) abdominal rumbling, (G) urgency, (H) abdominal pain (missing data *n* = 81). OATT stratified into groups representing: <25th percentile, most rapid OATT; 25–75th percentile, intermediate OATT; and >75th percentile, slowest OATT. Controlled for functional dyspepsia, anxiety, depression, and somatization. OATT, oroanal transit time. Created with BioRender.com.

The unadjusted analyses showed that more rapid OATT was associated with worse abdominal rumbling and urgency, with an additional OATT‐by‐time interaction effect for urgency, that is, urgency changed differently over time depending on OATT (Table 2). Although OATT was not associated with abdominal pain, a significant OATT‐by‐time interaction effect was found, indicating that OATT had no effect on abdominal pain itself, but the abdominal pain ratings changed differently over time depending on OATT. After adjusting for comorbid conditions, more rapid OATT was also found to be associated with worse abdominal discomfort and nausea (Table 2). Visualizations of the adjusted symptom ratings show that the symptoms nausea, urgency, abdominal discomfort, and rumbling are worse for those with more rapid OATT compared to those with slower OATT (Figure 1). Abdominal pain, urgency, and nausea ratings changed differently over time depending on OATT (Figure 1).

**TABLE 2:** Associations between oroanal transit time and postprandial symptoms in irritable bowel syndrome

	Gas	Bloating	Distension	Nausea	Discomfort	Rumbling	Urgency	Pain^c^
	*F*	*F*	*F*	*F*	*F*	*F*	*F*	*F*
	*β* (95% CI)	*β* (95% CI)	*β* (95% CI)	*β* (95% CI)	*β* (95% CI)	*β* (95% CI)	*β* (95% CI)	*β* (95% CI)
Unadjusted^a^								
Time	1.8*	3.1***	2.0**	3.7***	1.3	2.6***	3.3***	1.2
OATT	−0.02 (−0.06, 0.03)	0.02 (−0.03, 0.06)	0.03 (−0.01, 0.08)	−0.04 (−0.09, <0.01)	−0.02 (−0.06, 0.01)	−0.08 (−0.11, −0.03)***	−0.07 (−0.12, −0.02)***	0.05 (−0.01, 0.11)
Time‐by‐OATT	0.81	0.67	0.48	1.5	1.1	1.2	1.9*	1.9*
Adjusted^b^								
Time	1.8*	3.1***	2.1**	3.7***	1.5	2.6***	3.2***	1.2
OATT	−0.01 (−0.05, 0.03)	<−0.01 (−0.05, 0.04)	0.01 (−0.04, 0.06)	−0.06 (−0.11, −0.02)**	−0.05 (−0.08, −0.01)**	−0.09 (−0.13, −0.04)***	−0.08 (−0.13, −0.03)***	0.03 (−0.02, 0.08)
Time‐by‐OATT	0.89	0.65	0.47	1.8*	1.0	1.2	1.7*	1.8*

*Note*: Data are log‐transformed. In the unadjusted analyses, OATT (*n* = 254) has an effect on symptom scores for abdominal rumbling and urgency. In the adjusted analyses, OATT has an effect on symptom scores for abdominal discomfort, abdominal rumbling, nausea, and urgency. More rapid OATT is associated with worse symptom scores. Interaction effect with time is present for nausea (adjusted), urgency, and abdominal pain (both unadjusted and adjusted).

Abbreviation: OATT, oroanal transit time.

*p < 0.05; **p < 0.01; ***p < 0.001.

^a^ Linear mixed model with *β* coefficient, i.e., linear slope, and 95% CI for the main effect of OATT, and *F* test statistic for time (categorical variable) and interaction effect of OATT with time.

^b^ Linear mixed model with *β* coefficient, that is, linear slope, and 95% CI for the main effect of OATT, and *F* test statistic for time (categorical variable) and interaction effect of OATT with time, controlled for anxiety, depression, somatization, and functional dyspepsia.

^c^ Missing data (*n* = 81) due to methodological error.

### 3.4 Rectal sensitivity is associated with intensity of GI symptoms, but after adjusting for comorbid conditions solely with abdominal pain

**FIGURE 2:**
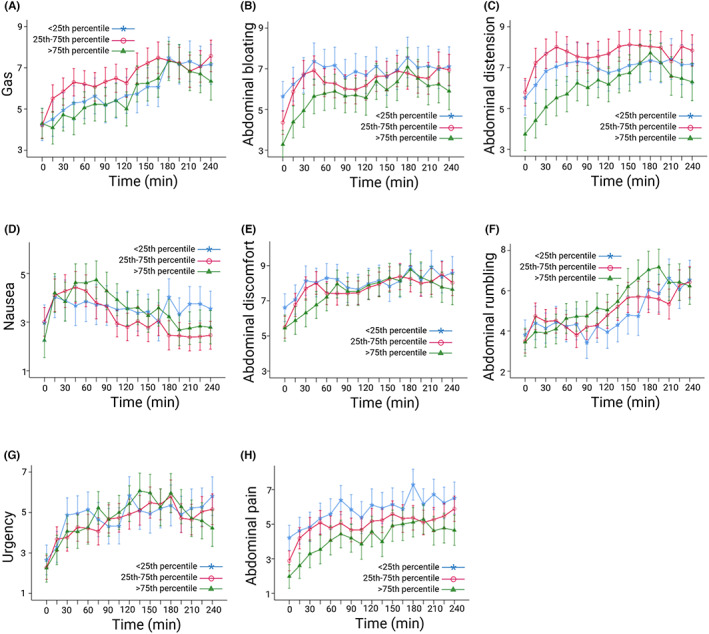
Associations between rectal sensitivity and postprandial symptom ratings in IBS patients (cohort 1; *n* = 129). The curves are estimated marginal means with standard error of the mean. (A) Gas, (B) abdominal bloating, (C) abdominal distension, (D) nausea, (E) abdominal discomfort, (F) abdominal rumbling, (G) urgency, (H) abdominal pain. Rectal sensitivity: pain threshold (mmHg) during rectal barostat, stratified into groups representing: <25th percentile, lowest rectal pain thresholds; 25–75th percentile, intermediate rectal pain thresholds; and >75th percentile, highest rectal pain thresholds. Controlled for functional dyspepsia, anxiety, depression, and somatization. Created with BioRender.com.

In the unadjusted analyses, lower rectal sensory thresholds were associated with worse nausea, abdominal bloating, discomfort, distension, and pain, and no interaction effects with time were observed. After controlling for comorbid conditions, solely abdominal pain was associated with lower rectal sensory thresholds (Table 3). The adjusted time‐symptom ratings for abdominal pain are highest for those with the lower rectal sensory threshold (Figure 2).

**TABLE 3:** Associations between rectal sensitivity and postprandial symptoms in irritable bowel syndrome

	Gas	Bloating	Distension	Nausea	Discomfort	Rumbling	Urgency	Pain
	*F*	*F*	*F*	*F*	*F*	*F*	*F*	*F*
	*β* (95% CI)	*β* (95% CI)	*β* (95% CI)	*β* (95% CI)	*β* (95% CI)	*β* (95% CI)	*β* (95% CI)	*β* (95% CI)
Unadjusted^a^								
Time	1.3	0.82	0.65	0.46	1.1	1.2	1.8 *	1.5
Rectal sensitivity	−0.01 (−0.01, 0.01)	−0.01 (−0.02, <−0.01)**	−0.01 (−0.02, <−0.01)*	−0.01 (−0.02, <−0.02)**	−0.01 (−0.02, <−0.01)**	−0.01 (0.01, <0.01)	−0.01 (−0.02, <−0.01)*	−0.01 (−0.02, −0.01)**
Time‐by‐rectal sensitivity	0.93	0.90	0.48	0.35	0.82	0.91	1.4	0.75
Adjusted^b^								
Time	1.2	0.88	0.62	0.44	1.0	1.2	1.8 *	1.4
Rectal sensitivity	<−0.01 (−0.01, 0.01)	−0.01 (−0.02, <0.01)	−0.01 (−0.01, <0.01)	−0.01 (−0.01, 0.01)	−0.01 (−0.01, <0.01)	<−0.01 (−0.01, 0.01)	−0.01 (−0.02, <0.01)	−0.01 (−0.02, <−0.01)*
Time‐by‐rectal sensitivity	0.88	1.1	0.55	0.34	0.81	0.96	1.5	0.75

*Note*: Data are log‐transformed. In the unadjusted analyses, rectal sensitivity (*n* = 129) has an effect on symptom scores for bloating, abdominal discomfort, abdominal distension, nausea, and abdominal pain. In the adjusted models, rectal sensitivity has an effect solely on abdominal pain. Lower rectal pain thresholds are associated with higher symptom scores.

*p < 0.05; **p < 0.01; ***p < 0.001.

^a^ Linear mixed model with *β* coefficient, that is, linear slope, and 95% CI for the main effect of rectal sensitivity, and *F* test statistic for time (categorical variable) and interaction effect of rectal sensitivity with time.

^b^ Linear mixed model with *β* coefficient, that is, linear slope, and 95% CI for the main effect of rectal sensitivity, and *F* test statistic for time (categorical variable) and interaction effect of rectal sensitivity with time, controlled for anxiety, depression, somatization, and functional dyspepsia.

### 3.5 Exhaled hydrogen/methane concentrations are associated with OATT, but not with rectal sensitivity

**FIGURE 3:**
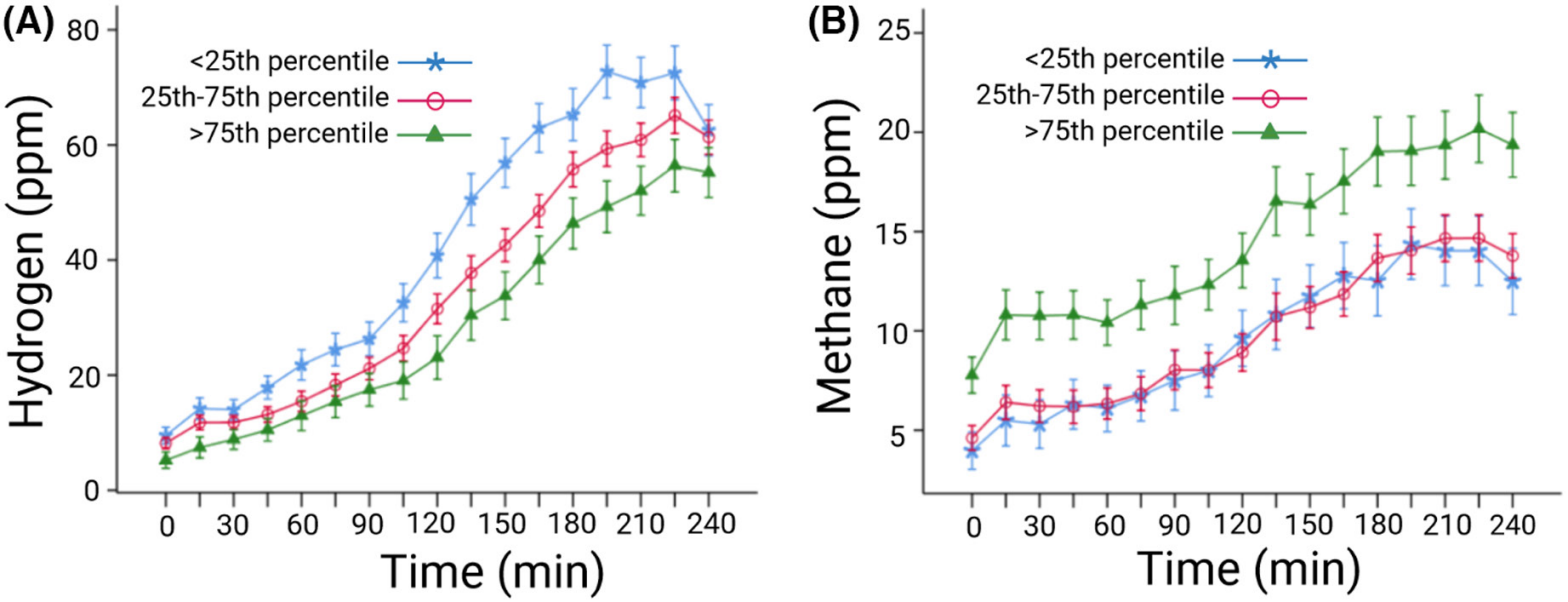
Associations between OATT and breath hydrogen and methane concentrations (parts per million) in IBS patients (*n* = 254). The curves are estimated marginal means with standard error of the mean. (A) Hydrogen, (B) methane. OATT stratified into groups representing: <25th percentile, most rapid OATT; 25–75th percentile, intermediate OATT; and >75th percentile, slowest OATT. OATT, oroanal transit time; ppm, parts per million. Created with BioRender.com.

OATT was negatively associated with exhaled hydrogen (OATT: *β* = −0.11 [−0.18, −0.04], *p* < 0.01; Time: *F* = 17.1, *p* < 0.001; Time‐by‐OATT interaction effect: *F* = 4.2, *p* < 0.05), indicating that more rapid OATT was associated with higher exhaled hydrogen concentrations, and hydrogen concentrations changed differently over time depending on OATT. OATT was positively associated with exhaled methane (OATT: *β* = 0.08 [0.03, 0.13], *p* < 0.01; Time: *F* = 11.8, *p* < 0.001; Time‐by‐OATT interaction effect: *F* = 0.16, *p* = 0.69), indicating that slower OATT was associated with higher exhaled methane concentrations. The curves of hydrogen are shifted upwards for those with more rapid OATT, and for methane, the curves are shifted upwards for those with slower OATT, and both hydrogen and methane generally increased over time (Figure 3). No associations between rectal sensitivity and hydrogen (Rectal sensitivity: *β* = <0.01 [−0.01, 0.01], *p* = 0.17; Time: *F* = 50.9, *p* < 0.001; Time‐by‐Rectal sensitivity interaction effect: *F* = 0.88, *p* = 0.59) or methane (Rectal sensitivity: *β* = <0.01 [−0.01, 0.01], *p* = 0.34; Time: *F* = 27.4, *p* < 0.001; Time‐by‐Rectal sensitivity interaction effect: *F* = 0.97, *p* = 0.49) were observed.

### 3.6 Postprandial symptoms and exhaled hydrogen/methane concentrations follow the same trajectory over time

**FIGURE 4:**
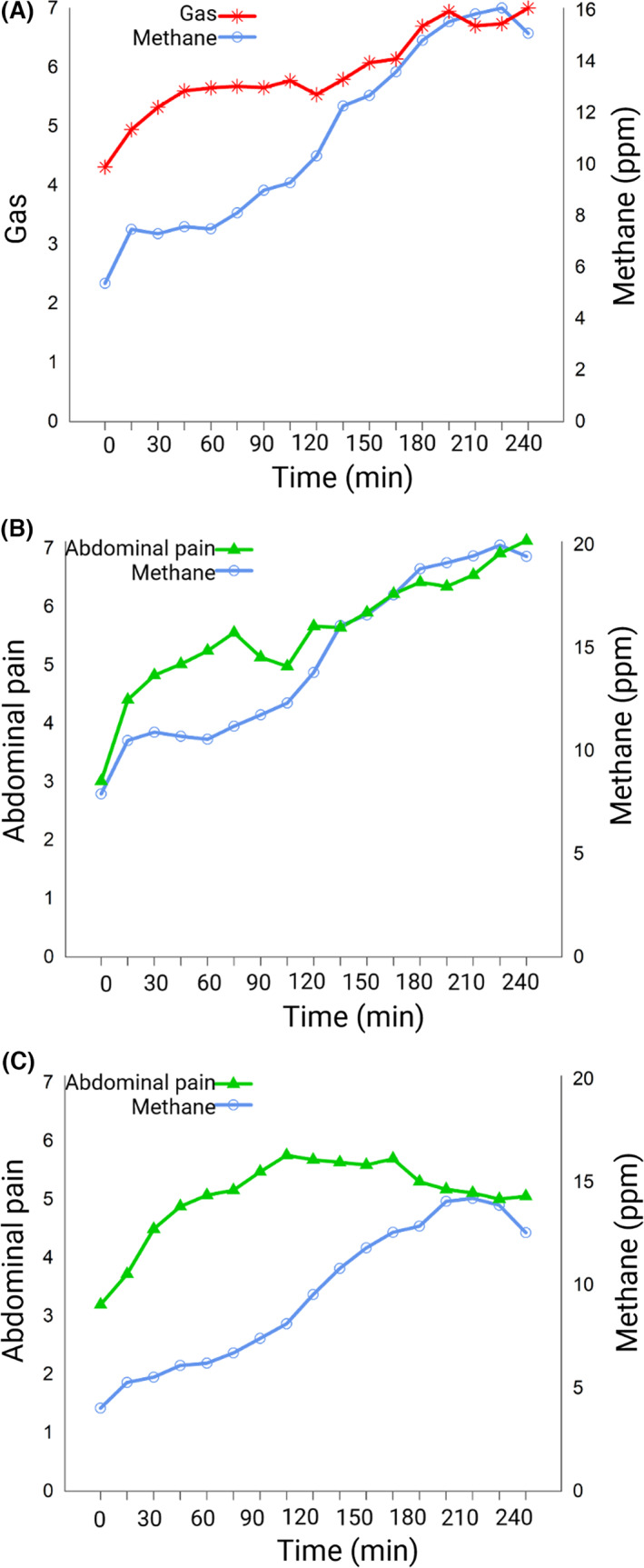
Trajectories over time (means) of postprandial symptom ratings and exhaled breath analyses (parts per million). (A) Postprandial gas ratings and exhaled methane concentrations in irritable bowel syndrome (*n* = 263), (B) postprandial pain ratings and exhaled methane concentrations in patients with slower OATT (<25th percentile, *n* = 46), (C) postprandial pain and exhaled methane concentrations in patients with more rapid OATT (>75th percentile, *n* = 46). OATT, oroanal transit time; ppm, parts per million. Created with BioRender.com.

Postprandial gas and abdominal rumbling ratings were dynamically correlated with exhaled hydrogen and methane (Table 4). The trajectories over time of postprandial gas and exhaled methane were plotted, and although the trajectory of methane was initially steeper compared to postprandial gas, the trajectories are similar. Both trajectories had an early rise, which stabilized and then increased again. After approximately 120 min, they followed the same trajectory (Figure 4A).

**TABLE 4:** Dynamical correlations^a^ between postprandial symptoms and exhaled hydrogen/methane concentrations in irritable bowel syndrome

	Hydrogen			Methane		
	WPC	*T*	*p*‐Value^b^	WPC	*T*	*p*‐Value^b^
Total cohort (*n* = 263)						
Gas	0.28	5.9	7.4E^−9^	0.28	6.6	9.4E^−11^
Bloating	0.06	−1.0	0.84	0.08	−1.2	0.89
Distension	0.06	0.70	0.25	0.14	1.5	1.4E^−4^
Nausea	−0.19	−4.8	1.0	0.13	3.7	1.0
Discomfort	0.15	2.4	0.01	−0.19	−4.1	0.06
Rumbling	0.27	2.5	7.0E^−3^	0.27	3.8	1.4E^−4^
Urgency	0.13	0.20	0.42	0.13	1.3	0.09
Pain (*n* = 182)	0.14	3.6	2.4E^−4^	0.16	4.0	4.8E^−5^
More rapid OATT (*n* = 59)						
Gas	0.22	2.1	0.02	0.17	2.0	0.03
Bloating	0.14	2.2	0.02	0.12	1.8	0.04
Distension	0.10	1.7	0.05	−0.05	−3.0	1.0
Nausea	−0.09	−0.50	0.69	0.01	−1.1	0.86
Discomfort	0.12	2.1	0.02	−0.11	−1.1	0.87
Rumbling	0.30	2.0	0.03	0.27	2.4	0.01
Urgency	0.13	0.01	0.49	0.11	0.01	0.49
Pain (*n* = 46)	−0.04	−2.8	1.0	−0.07	−2.7	1.0
Slower OATT (*n* = 59)						
Gas	0.32	2.6	5.0E^−3^	0.32	2.2	0.01
Bloating	0.02	−2.6	1.0	0.07	−3.6	1.0
Distension	0.15	−1.1	0.86	0.16	−1.7	0.95
Nausea	0.13	1.7	0.05	0.21	1.4	0.08
Discomfort	−0.29	−4.0	1.0	−0.20	−0.83	0.78
Rumbling	0.25	−2.3	0.99	0.23	−1.9	0.97
Urgency	0.07	−1.9	0.97	0.13	−0.74	0.76
Pain (*n* = 46)	0.33	4.2	8.0E^−5^	0.34	3.9	2.0E^−4^

Abbreviations: OATT, oroanal transit time; WPC, within‐person correlations.

^a^ Dynamical correlations are assessed simultaneously, that is, at the same pre‐ and post‐prandial time points. Interpretation of the dynamical correlations was done by the combination of WPC (0–1.0) and *p*‐values. Strong WPC in combination with *p* > 0.05 are irrelevant, as well as low *p*‐values in combination with weak WPC (<0.2).

^b^ *p*‐Values test the hypothesis that WPC are larger than regular between‐person (Pearson) correlations.

### 3.7 Different dynamical correlations between postprandial symptoms and exhaled hydrogen/methane concentrations depending on OATT

For those with more rapid OATT, postprandial gas was dynamically correlated with exhaled hydrogen, and postprandial abdominal rumbling was dynamically correlated with exhaled hydrogen and methane. For those with slower OATT, postprandial gas and abdominal pain were dynamically correlated with exhaled hydrogen and methane (Table 4). The trajectories over time of postprandial abdominal pain and exhaled methane were plotted for those with slower OATT and more rapid OATT. For those with slower OATT, postprandial abdominal pain and exhaled methane followed the same trajectory over time, with increasing trajectories for both abdominal pain and exhaled methane (Figure 4B), whereas for those with more rapid OATT, postprandial abdominal pain and exhaled methane did not follow the same trajectory over time (Figure 4C).

## 4 DISCUSSION

In this study, we demonstrated associations between OATT, hydrogen and methane production, and postprandial symptoms in IBS, which may be of importance for everyday symptoms in this large patient group and guide future therapeutic approaches. We showed that postprandial symptoms, hydrogen and methane production, and OATT are reciprocally associated in IBS patients, with different associations for those with more rapid and slower transit times, respectively. Moreover, several postprandial symptoms and exhaled hydrogen/methane followed the same trajectory over time, highlighting the correlation between hydrogen and methane production and postprandial symptoms.

Our results confirmed that anxiety, depression, somatization, and FD are independently associated with postprandial symptoms in IBS. Previous studies using LNCT and a meal challenge have shown these associations,^9^, ^10^ and the current study replicated these findings. For the unadjusted rectal sensitivity data, higher symptom ratings were associated with lower rectal pain thresholds, and these findings are in line with previous studies that have found associations between visceral hypersensitivity and the severity of GI symptoms in IBS.^5^ However, after controlling for the comorbid conditions, which are of importance for central nervous system function,^4^ solely abdominal pain remained significant in this cohort. This might be explained by the known associations between these conditions and visceral hypersensitivity. Both in IBS and FD, psychological factors strongly influence visceral sensitivity, and studies have shown that the psychological state is affecting symptom perception and reporting.^5^, ^25^, ^26^, ^27^ Therefore, the results indicate that not only central mechanisms, but also peripheral factors such as visceral sensory function may be involved in GI symptom generation.

The data showed that there was an immediate rise in symptoms following meal intake, especially for nausea, abdominal bloating, and urgency, that could be explained by both central and peripheral mechanisms. The patients were informed that the meal could induce symptoms, and the early rise in symptoms may include an element of anticipation, that is, a potential nocebo effect, which is common in IBS patients.^28^ Physiological responses of the gut were also to be expected. The patients were instructed to consume the lactulose‐nutrient drink within 15 min, thus consuming a high load of calories within a short time‐interval, which could induce an exaggerated sensory component of the gastrocolonic response.^29^ Other pathophysiological mechanisms may also be involved, for example, direct effects of food components on the gut mucosa causing a non‐IgE‐mediated immune response.^30^

A previous study from our group found that OATT and GI symptoms were poorly correlated in a large IBS population.^6^ Our current findings seem to contradict to this, as associations were observed between several GI symptoms and OATT. However, there are major differences between the two studies regarding symptom reports. In the previous study, solely three symptoms were investigated daily for 7 days, whereas in this study, eight symptoms were assessed at regular time intervals. Furthermore, we assessed symptoms after a provocative test meal including a non‐absorbable carbohydrate, where it can be expected that symptoms are induced in IBS.^8^ Several GI symptoms were associated with OATT, as well as different postprandial symptom responses over time depending on OATT. These associations indicate that different underlying mechanisms might be involved in symptom generation in IBS patients with different transit times. Our results indicate that GI motility could play a central role in generation of GI symptoms, and that treatments targeting transit time could improve pre‐ and postprandial symptoms.

Recent studies have highlighted the importance of the gut microbiota in IBS, and they produce intestinal gases through carbohydrate fermentation, which are proposed to be involved in symptom generation.^7^, ^14^ Studies in IBS populations have found that methane production was associated with hard stools, slower transit and constipation in general.^16^, ^31^, ^32^ Our results are in line with this, as we showed that patients with slower OATT have higher concentrations of methane than patients with more rapid OATT. Additionally, we showed that patients with more rapid OATT have higher hydrogen concentrations compared to patients with slower OATT. An earlier rise of hydrogen is to be expected in patients with more rapid transit, due to the effects of lactulose. However, the finding that the hydrogen rise is generally higher with more rapid OATT could not solely be explained by the effect of lactulose. We hypothesize that differences in substrate exposure time after reaching the colon related to individual differences in colonic transit time may be a contributing factor of importance. Furthermore, different dynamical correlations were found in patients with slower and more rapid OATT, respectively. Postprandial abdominal pain was correlated with exhaled hydrogen/methane concentrations with similar trajectories over time in patients with slower OATT, but not in patients with more rapid OATT. Thus, these findings indicate that there may be a common relationship at play and highlight the importance of the gut microenvironment in IBS in relation to OATT and to postprandial symptom generation. We hypothesize that IBS patients with more rapid and slower OATT have different gut microbial compositions, producing different gases and metabolites, and could therefore have different underlying mechanisms in symptom generation.

Previous studies found that (postprandial) GI symptoms were not associated with hydrogen and methane production,^8^, ^15^, ^16^, ^30^ but we showed that ratings of several postprandial symptoms and exhaled hydrogen/methane concentrations over time were correlated. An explanation for this may be the different statistical approach that was used in the current study. The previous studies converted variables with multiple parameters to variables with solely one parameter, with loss of valuable data,^8^, ^15^, ^16^, ^31^ whereas the current study used dynamical correlations, which utilizes all parameters. Our results showed that especially postprandial gas and abdominal rumbling were dynamically correlated with exhaled hydrogen/methane concentrations, which indicates that postprandial symptoms and hydrogen and methane production could be related. We hypothesize that ingestion of a meal containing lactulose leads to increased hydrogen and methane production, which leads to simultaneously increased subjective postprandial gas and abdominal rumbling reports. The pathophysiological mechanisms of the other symptoms are more complex and multifactorial, including the expectation of symptoms and osmotic effects in the small intestine.^28^, ^33^, ^34^ The linear mixed model analyses showed that those with more rapid versus slower OATT had different productions of hydrogen and methane. Therefore, we decided to analyze the dynamical correlations between postprandial symptoms and hydrogen/methane production separately for these groups. As expected, the results were similar for gas and abdominal rumbling for both groups, but we observed differences for abdominal pain. For those with slower OATT, abdominal pain and both hydrogen and methane followed the same trajectory over time. However, there were no dynamical correlations for those with more rapid OATT. We hypothesize that hydrogen and methane production may be the driver of abdominal pain for those with slower OATT, but not for those with more rapid OATT.

Strengths of this study include that we reproduced and confirmed previous associations between postprandial symptoms and comorbid conditions in a large IBS population. The adjusted analyses on OATT and rectal sensitivity were controlled for these important features in IBS. The study population was well‐characterized and exclusively validated questionnaires and clinical/research investigations were used. All patients were studied in a single expert center for neurogastroenterology, minimizing differences in methodology, and the aims of both cohorts were similar. A limitation is that this was a retrospective study which warrants for caution regarding concluding causality of the associations. We also combined data from two cohorts where different versions of the Rome criteria were used for inclusion, and the Rome IV criteria identify a more severe group of IBS patients.^35^ However, this could also be considered as a strength since our data show no differences in characteristics between the cohorts. Another limitation is that we solely assessed hydrogen and methane production but no other gases, for example, carbon dioxide and hydrogen sulfide, and metabolites, for example, short‐chain fatty acids, that are of importance for gut physiology.^36^ It remains unclear whether other gases and metabolites produced by the gut microbiota and other metabolic processes in the gut could be associated with postprandial symptoms and transit time in IBS. Due to a methodological error, abdominal pain data of the LNCT were not reported by 81 patients in cohort 2. Therefore, the LNCT results of abdominal pain should be interpreted with caution. In addition, rectal sensitivity was solely assessed in cohort 1 and a higher number of patients would have yielded more robust estimates, but the abdominal pain results are valid because there was no missing data regarding this in cohort 1.

In conclusion, our results show that OATT is associated with the severity of GI symptoms, specific postprandial symptom responses, and hydrogen and methane production. Our findings indicate that the assessment of OATT could be useful in IBS patients with postprandial symptoms. Moreover, OATT abnormalities provide an objective treatment target for clinicians. These findings increase our understanding of pathophysiological factors involved in postprandial symptom generation, highlight the relevance of transit time and hydrogen and methane production in the pathophysiology of IBS, and suggest the validity of these as relevant treatment targets for reduction of postprandial symptoms.

## AUTHOR CONTRIBUTIONS

All authors had access to the study data and reviewed and approved the final manuscript. All co‐authors involved in writing—review and editing. Joost P. Algera, Egbert Clevers, and Jóhann P. Hreinsson involved in data curation, formal analysis, software, and visualization. Joost P. Algera involved in conceptualization, methodology, and writing—original draft. Magnus Simrén and Hans Törnblom involved in study supervision, data collection, investigation, resources, and funding acquisition.

## FUNDING INFORMATION

The study was financed by grants from the Swedish state under the agreement between the Swedish government and the county councils, the ALF‐agreement (ALFGBG‐726561, 722331, 875581), the Swedish Research Council (2018–02566), and the Faculty of Medicine at the University of Gothenburg (Dnr DS2019/1843).

## CONFLICT OF INTEREST

Joost P. Algera, Esther Colomier, Jóhann P. Hreinsson, Irina Midenfjord, Egbert Clevers, and Hans Törnblom have no conflicts of interest to declare. Chloé Melchior has served as a consultant/advisory board member for Kyowa Kirin, Norgine, Biocodex, MayolySpindler, Tillots, and Ipsen. Magnus Simrén has received unrestricted research grants from Glycom, and served as advisory board member/consultant and/or speaker for Danone Nutricia Research, Ironwood, Menarini, Biocodex, Genetic Analysis AS, DSM, Tillotts, Takeda, Arena, Kyowa Kirin, Adnovate, Biocodex, AlfaSigma, Sanofi, Janssen Immunology, Pfizer, Falk Foundation, and Atnahs Pharma.

## Supporting information


Appendix S1
