# Supplementary material for: Associations between postprandial symptoms, hydrogen and methane production, and transit time in irritable bowel syndrome
Source: Neurogastroenterol Motil. 2022 Oct 11;35(2):e14482. doi: 10.1111/nmo.14482 (PMC10078221; doi:10.1111/nmo.14482)
Supplement: Supplementary file 1 — Appendix S1 [file NMO-35-0-s001.pdf]

Supplements to

Associations between postprandial symptoms, hydrogen and methane production and transit time in irritable bowel syndrome

Algera JP, et al.

Contents

Supplementary methods page 2

Supplementary figure page 3

Supplementary tables page 4, 5

SUPPLEMENTARY METHODS

Dynamical correlations

Stepwise approach of dynamical correlations

1. Values of every participant were min/max-normalized. Arrays with only one unique value were converted to missing.
2. Within-person correlations were computed between all pairs of variables, i.e. based on 17 time points. The median within-person correlations of n subjects were noted.
3. To generate a null-distribution, values of each time point were shuffled randomly across participants, and the median within-person correlations were recomputed. This was done 100,000 times. This null-distribution reflects the null hypothesis that within-person correlations are as large as regular between-person (Pearson) correlations.
4. The within-person correlation minus the mean of the null distribution was divided by the standard deviation of the null distribution. This was taken as T value.
5. One-tailed P values were computed from the T values in conjunction with the degrees of freedom (n -1).
6. The process was also performed with subgroups of oroanal transit time.

See **Supplementary figure 2** for more details on within-person and between-person correlations, as well as interpretation of trajectories over time.

SUPPLEMENTARY FIGURE

Supplementary figure 1. Study planning. LNCT, lactulose nutrient challenge test; OATT, oroanal transit time; V, visit. *Created with BioRender.com.*

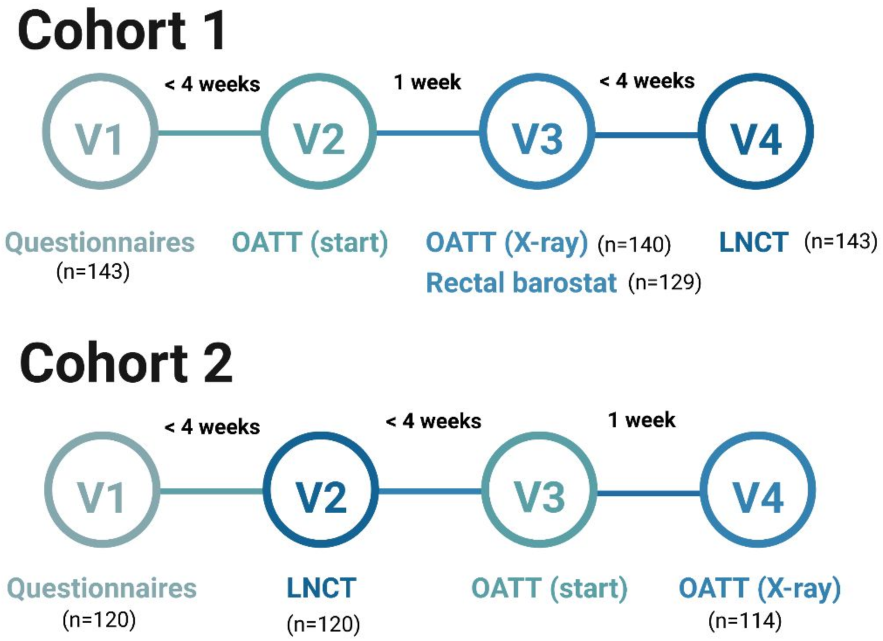

Supplementary figure 2. Illustrations of relationships within-person and between person correlations

(A) Although exhaled gas concentration and the postprandial symptom follow a common trajectory, these may both be caused by the intervention as such. Causality inference between exhaled gas and the postprandial symptom would be unfounded.
(B) Exhaled gas concentration and the postprandial symptom follow a common trajectory within the individual. There may be a common process at play.
(C) No correlation found between exhaled gas concentration and postprandial symptom.
(D) No correlation found between exhaled gas concentration and postprandial symptom.

Created with BioRender.com.

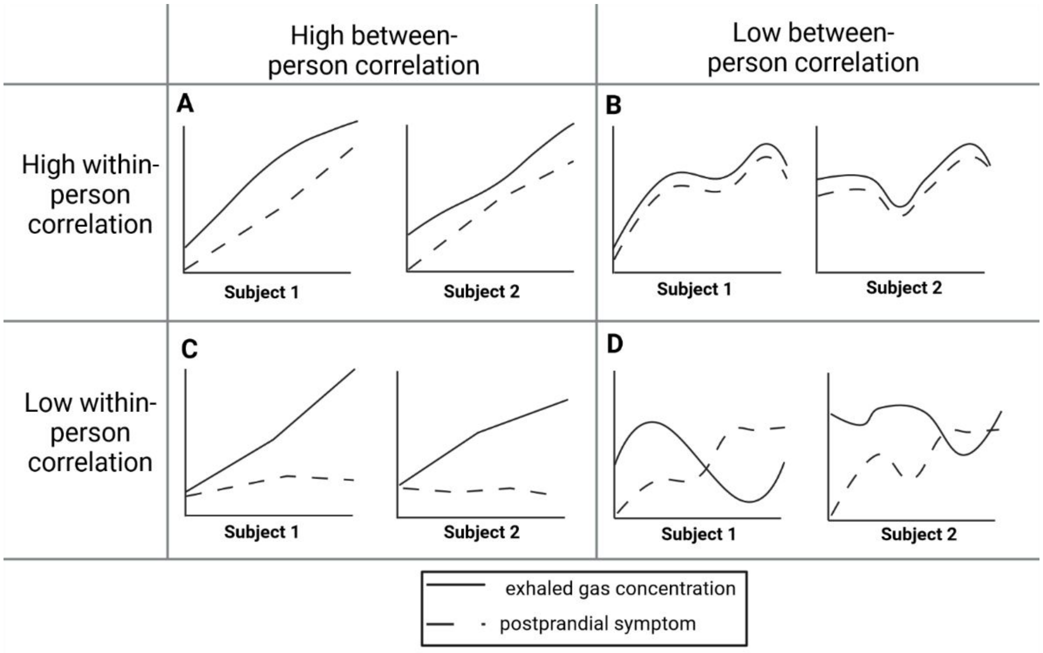

SUPPLEMENTARY TABLES

Supplementary table 1. **Assessment of collinearity between the variables**

	Spearman correlation coefficients					Eta squared correlations
	Anxiety	Depression	Somatization	Oroanal transit time	Rectal pain threshold (cohort 1)	Functional dyspepsia
Anxiety		.56 ‡	.48 ‡	.08	-.19 †	.19
Depression			.46 ‡	.07	<.01	.17
Somatization				.15 †	-.23 †	.21 †
Oroanal transit time					-.09	.03
Rectal pain threshold (cohort 1)						.15
Functional dyspepsia						

† weak correlation; ‡ moderate correlation. No collinearity was observed.

Supplementary table 2. Associations between postprandial symptoms and anxiety, depression, somatization, and functional dyspepsia in IBS†

	Gas	Bloating	Distension	Nausea	Discomfort	Rumbling	Urgency	Pain ‡
	F<br>β (95% CI)	F<br>β (95% CI)	F<br>β (95% CI)	F<br>β (95% CI)	F<br>β (95% CI)	F<br>β (95% CI)	F<br>β (95% CI)	F<br>β (95% CI)
Time	2.64 ***	3.32 ***	2.01 **	1.65 *	.94	1.89 *	1.41	.90
Anxiety	.01 (.0001, .02)*	.02 (.01, .03) **	.01 (.004, .02) **	.02 (.01, .03) ***	.02 (.01, .02) ***	.01 (.001, .02) *	.01 (-.001, .02)	.02 (.01, .04) ***
Time-by-anxiety	.93	1.13	.82	.41	.44	.65	.63	1.29
Time	2.53 ***	4.55 ***	2.94 ***	2.73 ***	2.08 **	2.26 **	2.53 ***	1.93 *
Depression	.01 (-.001, .02)	.02 (.01, .03) ***	.02 (.01, .03) **	.02 (.01, .03) ***	.02 (.01, .03) ***	.01 (-.004, .02)	.02 (.004, .03) *	.03 (.01, .05) ***
Time-by-depression	.88	.99	.47	.56	.55	1.01	1.10	2.18 **
Time	2.27 **	3.37 ***	2.71 ***	2.35 **	2.01 **	1.70 *	1.74 *	1.36
Somatization	.01 (-.01, .02)	.02 (.01, .03) **	.02 (.01, .03) ***	.02 (.01, .03) ***	.02 (.01, .03)	.01 (-.0001, .02)	.01 (-.002, .02)	.04 (.03, .06)
Time-by-somatization	1.13	1.32	1.36	.98	.83	.45	1.28	1.35
Time	4.85 ***	7.39 ***	6.12 ***	7.26 ***	4.33 ***	3.92 ***	5.60 ***	3.70 ***
FD	-.10 (-.19, -.01)*	-.13 (-.23, -.04) **	-.13 (-.23, -.04)	-.14 (-.24, -.05) **	-.12 (-.19, -.04) **	-.06 (-.15, .03)	-.10 (-.20, .01)	-.30 (-.42, -.17) ***
Time-by-FD	1.04	.80	.84	1.11	.73	.82	.50	.70

CI, confidence interval; FD, functional dyspepsia; HADS, Hospital Anxiety and Depression Scale; IBS, irritable bowel syndrome; PHQ, Patient Health Questionnaire.

Data are log-transformed. Significant positive β coefficients demonstrated that increasing values of the independent variables (or absence of FD) were associated with worse postprandial symptom severity. Significant negative β coefficients demonstrated that decreasing values of the independent variables (or presence of functional dyspepsia) were associated with worse postprandial symptom severity. A significant interaction effect indicated that the postprandial symptom changed differently over time, depending on the independent variable, e.g., postprandial abdominal pain changed differently over time depending on level of depression.

† Linear mixed models presented as β coefficient, i.e., linear slope, and 95% CI for the main effect of the continuous and dichotomous variables, and F test statistic for time (categorical variable) and the interaction effect with time.

‡ Missing data (n=81) due to methodological error

* p<.05; ** p<.01; *** p<.001.
